# The transcription factor RBP-J is essential for retinal cell differentiation and lamination

**DOI:** 10.1186/1756-6606-2-38

**Published:** 2009-12-18

**Authors:** Min-Hua Zheng, Ming Shi, Zhe Pei, Fang Gao, Hua Han, Yu-Qiang Ding

**Affiliations:** 1 Department of Medical Genetics and Developmental Biology, Fourth Military Medical University, Xi'an 710032, China; 2 Department of Anatomy and Neurobiology, Tongji University School of Medicine, Shanghai 200092, China; 3 Present address: Department of Neurology, Xijng Hospital, Fourth Military Medical University, Xi'an 710032, China

## Abstract

**Background:**

The highly ordered vertebrate retina is composed of seven cell types derived from a common pool of retinal progenitor cells (RPCs), and is a good model for the studies of cell differentiation and interaction during neural development. Notch signaling plays a pivotal role in retinogenesis in mammals, but the full scope of the functions of Notch pathway, and the underlying molecular mechanisms, remain unclear.

**Results:**

In this study, we conditionally knocked out *RBP-J*, the critical transcription factor downstream to all four Notch receptors, in RPCs of mouse retina at different developmental stages. Disruption of RBP-J at early retinogenesis resulted in accelerated RPCs differentiation, but only photoreceptors and ganglion cells were overrepresented, with other neuronal populations diminished. Similarly, deletion of *RBP-J *at early postnatal days also led to overproduction of photoreceptors, suggesting that RBP-J governed RPCs specification and differentiation through retinogenesis. In all the *RBP-J *deletion models, the retinal laminar structures were distorted by the formation of numerous rosette-like structures, reminiscent of β-catenin deficient retina. Indeed, we found that these rosettes aligned with gaps in β-catenin expression at the apical surface of the retina. By in vivo electroporation-mediated transfection, we demonstrated that lamination defects in *RBP-J *deficient retinae were rescued by overexpressing β-catenin.

**Conclusions:**

Our data indicate that RBP-J-mediated canonical Notch signaling governs retinal cell specification and differentiation, and maintains retinal lamination through the expression of β-catenin.

## Background

The vertebrate retina consists of seven cell types organized into distinct laminar structures. In mice, retinal neurogenesis begins at embryonic (E) day 11.0 and terminates around postnatal (P) day 11. The six neuronal cell types and one glial cell type that make up the retina all derive from common retinal progenitor cells (RPCs) [1]. RPCs give rise to retinal cells in a conserved chronological sequence: ganglion cells and horizontal cells are born first, followed by cone photoreceptors and amacrine cells during the middle stage of retinogenesis. Rod photoreceptors, bipolar cells and Müller glial cells are the last cell types to be generated, mainly during postnatal stages [2].

The retina has been serving as an excellent model for the studies of cell differentiation and interaction during neural development, attributing to its limited cell types generated in a temporal-spatially defined process. Among molecules and pathways involved in retinal development, the Notch signaling has been demonstrated as an essential regulator of retinogenesis [3,4]. In mice, the canonical Notch pathway includes five ligands (Delta-like [Dll] 1, 3, 4, Jagged1, 2), four receptors (Notch1-4), and the transcription factor recombination signal-binding protein Jκ (RBP-J) (also termed CBF1 in mammals), which regulates the expression of downstream genes such as the Hairy and enhancer of split (*Hes*) family members. Both Notch ligands and receptors are type I transmembrane proteins mediating direct cell-cell interactions. Upon ligand binding, the Notch intracellular domain (NICD) is released by proteolytic cleavages within the transmembrane domain, and translocates into the nucleus, where it interacts with RBP-J and transactivates the transcription of downstream genes. Because RBP-J binds to NICD of all four mammalian Notch receptors, it serves as the key integrator of canonical Notch signaling [5,6].

Many members of this cascade have been shown to regulate retinogenesis. Transfection of an activated form of *Xotch *(the homologue of *Notch *in *Xenopus*) into cultured *Xenopus *retinal cells retains their neuroepithelial morphology [7]. Injection of antisense oligoneucleotides of *CNotch1 *(the chicken *Notch1 *homologue) into undifferentiated chicken retina increases the recruitment of RPCs differentiating into ganglion cells [8]. *Notch1 *deficient retinae have more cone photoreceptors and less ganglion cells [9,10], whereas in *Hes1 *mutant mice, ganglion cells are overproduced [11]. Deleting *Hes5 *in the mouse retina significantly decreases Müller glial cell population [12]. These findings while revealing the importance of Notch signaling in retinal cell specification and differentiation, demonstrate that the manipulations of individual genes in the Notch pathway can yield a variety of phenotypes, and therefore highlight a need of further studies to fully understand the complexity and molecular mechanisms of Notch signaling during retinal development.

While adopting specific cell fates, differentiating retinal cells migrate to appropriate laminae during retinogenesis. RPCs orient radially along the apical/basal axis and extend their endfeet anchored on either side [13,14]. Retinal cell migration requires the detachment of their endfeet from the apical adherens junctions [15,16]. Retina-specific inactivation of β-catenin, a component of adherens junctions, has been shown to result in severe retinal lamination defects without affecting cell specification or differentiation [17], suggesting the importance of cell adhesion in retinal lamination. Interestingly, eliminating *Notch1 *expression from the developing retina also leads to severe lamination defects [9,10], although the underlying molecular mechanisms remain to be elusive.

To gain more insight into the various functions of the Notch signaling pathway in retinal development, we conditionally deleted *RBP-J *in the mouse retina at both embryonic and postnatal stages. Our results show that RBP-J-mediated canonical Notch signaling not only governs retinal cell specification and differentiation, but also maintains retinal lamination as well, which is achieved probably through the expression of β-catenin.

## Methods

### Animals

Mice were maintained on the C57BL/6 genetic background. Mice carrying the Chx10Cre BAC [18] or Pet1Cre BAC [19] transgene were crossed with mice carrying a floxed *RBP-J *allele [5] to obtain *Chx10Cre-RBP*^*f*/*f *^mice or *Pet1Cre-RBP*^*f*/*f *^mice. Mice bearing either of these two genotypes survived into adulthood. The expression of Cre in the neural retina of *Pet1Cre *mice was determined by X-gal staining after crossing to Rosa26 reporter mice [20]. Animal experiments were reviewed and approved by the Animal Experiment Administration Committee of the Fourth Military Medical University.

### Immunohistochemistry and in situ hybridization

Tissues were fixed overnight in 4% paraformaldehyde in 0.1 M phosphate buffer (PB; pH 7.4) at 4°C. After cryoprotection with 15% sucrose in PB, 14 μm-thick sections were cut on a cryostat and mounted onto polylysine-coated glass slides. For immunostaining, sections were hydrated in 0.01 M phosphate-buffered saline (PBS; pH 7.4), blocked in PBS containing 1% donkey serum and 0.1% Triton X-100 for 2 hours, and incubated with primary antibodies overnight at 4°C. The primary antibodies used were: mouse anti-BrdU (1:200; Calbiochem), rabbit anti-EGFP (1:2000; Molecular Probes), goat anti-Brn3 (1:200; Santa Cruz), mouse anti-Neurofilament 165 KDa (NF165; 1:500; Hybridoma Bank), mouse anti-syntaxin (1:1000; Sigma), rabbit anti-NK3, rabbit anti-PKCα (1:600; Santa Cruz), mouse anti-rhodopsin (1:500; Chemicon), rabbit anti-glutamine synthetase (1:500; BD Pharmingen), and rabbit anti-GFAP (1:1000; Dako Cytomation). After washing in PBS, immunoreactivity was detected using Cy3 or Cy2-conjugated secondary antibodies (1:200; Jackson ImmunoResearch). For detection of cone photoreceptors, fluorescein-conjugated peanut agglutinin (PNA; 1:200; Vector) was employed. Sections were counterstained with Hoechst (Sigma) and coverslipped with 20% glycerol in PBS.

*In situ *hybridization was performed in essentially the same manner as described by Guo et al. [21]. The following mouse antisense RNA probes were used: *neurofilament light polypeptide *(*Nefl*), *Islet1*, *clusterin *[9], *Gnat1*, *Arr3 *[22], *CyclinD1*, *Fgf15 *[23], *Math5*, *Neurod1*, *Ngn2, Mash1 *[24], *Math3 *[25], *Crx*, *Thrβ2 *[10], *Delta-like1 *(*Dll1*), *Pax6 *[26], *Sox2 *[27], *Hes1 *[26], *Hes5 *[12], and *Hesr2 *[28]. The *RBP-J *(0.3 kb for exons 6 and 7; GenBank accession number: NM-009035), *Notch1-4 *(NM-008714, NM-010928, NM-008716, NM-010929, respectively), *Jagged1 *(NM-013822), *Otx2 *(NM-144841) and *Ptf1a *(NM-018809) probes were amplified by PCR using cDNA templates prepared from E14.5 mouse embryos. Hematoxylin-Eosin (HE) and Nissl staining were performed according to standard protocols.

### BrdU labeling and terminal deoxynucleotidyl transferase-mediatd biotinylated UTP nick end labeling (TUNEL)

Pregnant mice were given a single intraperitoneal injection of BrdU (60 μg/g body weight) at 12.5 and 13.5 days postcoitum, and were sacrificed 2 hours later. Sections were processed for immunostaining with anti-BrdU, as described above. TUNEL assay was performed using the In Situ Cell Death Detection Kit (Roche).

### DNA constructs

For the construction of pCAG-β-catenin, full length β-catenin cDNA [29] was inserted into the pCAG vector. pCAG-Cre and pCAG-GFP plasmids were generous gifts from Dr. Constance L. Cepko [30]. Plasmid DNA was purified using QIAfilter Plasmid Midi and Maxi Kits (Qiagen), and injected at a concentration of 1 μg/μl.

### *In vivo *electroporation

In vivo electroporation of P0 and P5 mouse pups was performed as described [30]. Five square pulses of 50 milliseconds duration with 1 second intervals were applied using a BTX ECM830 pulse generator (Boston, MA, USA). Pulses with 80 V and 85 V were applied to P0 and P5 pups, respectively. pCAG-EGFP was coelectroporated to visualize transfected cells, and immunostaining was performed to confirm expression of electroporated genes.

### Statistics

Cells were counted with Stereo Investigator software (MBF Bioscience), and only cell bodies that were labeled with immunoreactivity were included. Proportions of immunoreactive cells in the total population of retinal cells revealed by Hoechst staining or in total electroporated cells labeled by EGFP or Cre were calculated, and comparisons were made using unpaired Student's *t*-test. Three sections through the optic disc were selected from each eye, and at least four eyes from each genotype were analyzed. Data were expressed as mean ± s.e.m.

## Results

### Reduced eye size and disrupted laminar structure of *RBP-J *deficient retinae

**Figure 1:**
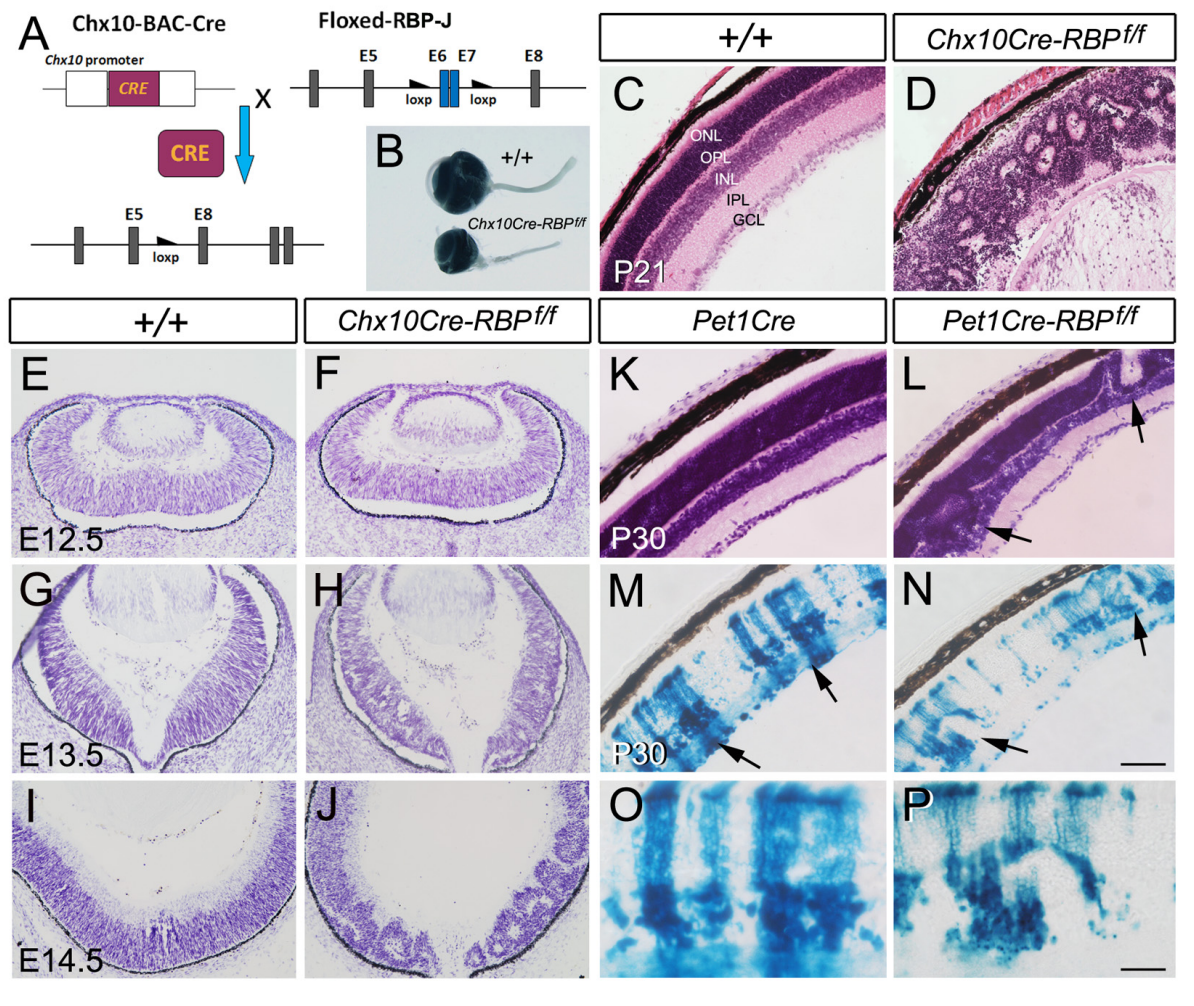
**Reduced eye size and disrupted lamination in *Chx10Cre-RBP^*f*/*f *^*retinae**. **(A) **Schematic illustration depicting the genetic scheme used to generate *Chx10Cre-RBP^*f*/*f *^*mice. **(B) **P21 *Chx10Cre-RBP^*f*/*f *^*mice show reduced eye size relative to wild-type controls. **(C, D) **HE staining of P21 retinal sections shows three nuclear layers and two synaptic layers are distinctly recognizable in wild-type mice (C), but the lamination is severely distorted with many rosettes in *Chx10Cre*-*RBP*^*f*/*f *^retina (D). **(E-J) **Nissl staining of wild-type (E, G, I) and *Chx10Cre*-*RBP*^*f*/*f *^retinae (F, H, J) at E12.5 (E, F), E13.5 (G, H), and E14.5 (I, J). Note that the rosettes are first detected at E13.5. **(K-N) **The adjacent sections processed respectively for HE and X-gal staining indicate that the clustered X-gal^+ ^(mutant) cells (arrows) are located within the rosettes in *Pet1Cre-RBP*^*f*/*f *^retinae (L, N). Images from *Pet1Cre *retinae are shown for comparison (K, M). O and P are high magnification views of (M) and (N), respectively. GCL, ganglion cell layer; INL, inner nuclear layer; IPL, inner plexiform layer; ONL, outer nuclear layer; OPL, outer plexiform layer. Scale bar, C-N, 100 μm, O, P, 50 μm.

We first used *in situ *hybridization to determine the expression of *RBP-J *in the developing retina. *RBP-J *transcripts were first detected in the neural retina at E10.5, at higher levels at E11.5 and E13.5. During this period, the transcripts of Notch receptors (*Notch1, 3*) and ligands (*Dll1, Jagged1*) were also detected (Additional file 1: Figure S1 and data not shown). In addition, *RBP-J *transcripts were still detectable in the retina at E16.5 and early postnatal stages (P0 and P5; data not shown). To study the putative roles of RBP-J in retinal development, we generated *Chx10Cre-RBP*^*f*/*f *^mice (Figure 1A) using RBP-J-floxed mice [5] and Chx10Cre mice [18], in which Cre expression was started in RPCs at approximately E10.5, resulting in almost complete loss of *RBP-J *in the retinae by E13.5 (Additional file 1: Figure S2). Compared to wild-type controls, the eyes of *Chx10Cre-RBP*^*f*/*f *^mice were greatly reduced in size at postnatal stages (Figure 1B). HE staining at P21 showed that in *Chx10Cre-RBP*^*f*/*f *^mice, the cellular organization of the laminar retinal structure was totally distorted by the appearance of rosette-like structures (Figure 1C and 1D). This is similar to that previously reported in *Notch1 *deficient retinae [9].

We next examined *Chx10Cre-RBP*^*f*/*f *^eyes at various embryonic stages to determine the time of onset of these morphological abnormalities. The results showed that rosette-like structures were detectable as early as E13.5 (Figure 1E-J), while eye size was notably reduced by E14.5 (data not shown).

*Pet1Cre *mice [19] express Cre in the retina as of E11.0. In contrast to the nearly ubiquitous Cre expression in *Chx10Cre *mice, Cre activity in *Pet1Cre *mice was patchy and restricted to a small portion of retinal cells (Additional file 1: Figure S3 A-D). *Pet1Cre-RBP*^*f*/*f *^mice did not show lamination defects at embryonic stages, although a few rosettes were found at P30 (Figure 1K and 1L). To clarify the relationship between rosettes and *RBP-J *mutant cells, we crossed *Pet1Cre-RBP*^*f*/*f *^mice to Rosa26 reporter mice and observed the distribution of X-gal^+ ^cells at P21. Interestingly, X-gal^+ ^(mutant) cells were clustered in these rosettes (Figure 1M-P), suggesting that the lamination defects observed in *RBP-J *deficient retina is likely a cell-autonomous effect of the loss of *RBP-J*. Taken together, our data indicate that inactivation of *RBP-J *expression in the retina results in a severe reduction of eye size and abnormal lamination during retinal development.

### Decreased cell proliferation and increased apoptosis in *RBP-J *deficient retinae

**Figure 2:**
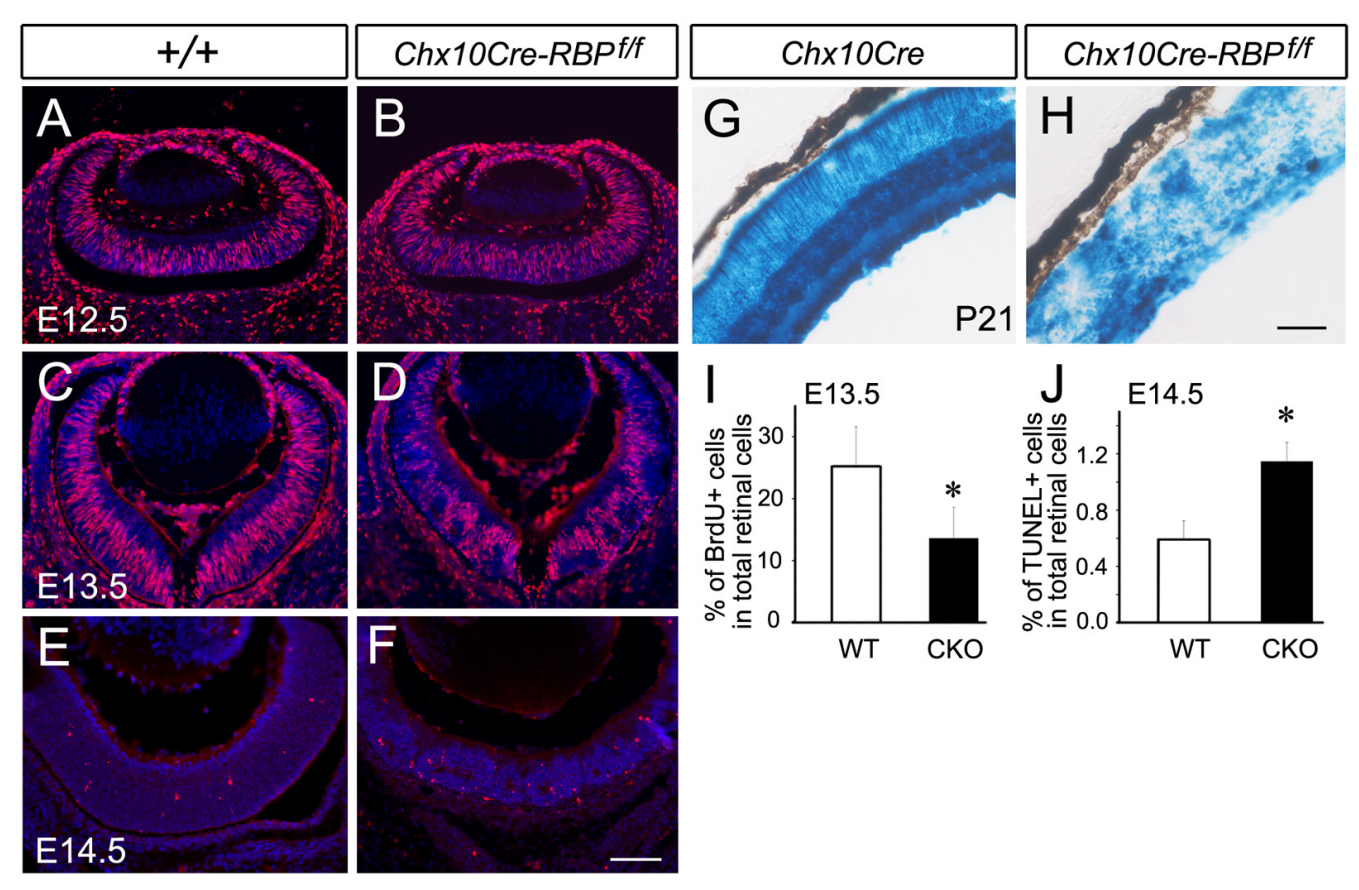
**Decreased cell proliferation and increased apoptosis in *Chx10Cre-RBP*^*f*/*f *^retinae**. **(A-D) **BrdU labeling shows reduced cell proliferation in *Chx10Cre-RBP*^*f*/*f *^retina at E13.5 (D), but not at E12.5 (B), relative to wild-type controls (A, C). **(E, F) **TUNEL staining reveals increased cell death in *Chx10Cre-RBP*^*f*/*f*^retina at E14.5 (F) compared to wild-type controls (E). **(G, H) **X-gal^+ ^mutant cells in *Chx10Cre-RBP*^*f*/*f *^retinae predominated and were distributed throughout the retina, albeit less uniformly (H), compared to that in *Chx10Cre *retinae (G). **(I, J) **Significant difference of percentages of BrdU^+ ^cells in the total retinal cells is observed between the two genotypes at E13.5 (I, 25.3 ± 6.3% of the total retinal cells in wild-type, 13.6 ± 4.9% of the total retinal cells in *Chx10Cre-RBP*^*f*/*f*^; *P *< 0.05), and significant difference of TUNEL^+ ^cells is found at E14.5 (J, 0.59 ± 0.13% of the total retinal cells in wild-type, 1.15 ± 0.14% of the total retinal cells in *Chx10Cre-RBP*^*f*/*f*^; *P *< 0.05). Scale bars, 100 μm.

To determine whether these defects were due to changes in cellular proliferation and/or apoptosis, we performed BrdU and TUNEL labeling between E12.5 and E14.5. BrdU labeling showed significantly reduced cell proliferation at E13.5, while TUNEL assay revealed an increase in the percentage of apoptotic cells at E14.5 in *Chx10Cre-RBP*^*f*/*f *^retinae compared to controls (Figure 2A-F, I and 2J).

The reduced proliferation and enhanced apoptosis in E13.5-E14.5 *Chx10Cre-RBP*^*f*/*f *^eyes raised the possibility that *RBP-J *mutant cells were diminished at later stages of development. To clarify this, we crossed *Chx10Cre-RBP*^*f*/*f *^mice to Rosa26 reporter mice and observed the distribution of X-gal^+ ^cells at P21. In *Chx10Cre *retinae, X-gal^+ ^cells constituted the vast majority of retinal tissue and were homogeneously present throughout the retina (Figure 2G). Similarly, in *Chx10Cre-RBP*^*f*/*f *^mice, X-gal^+ ^cells predominated and were distributed throughout the retina, albeit less uniformly (Figure 2H), showing that although increased cell death occured in *RBP-J *deficient retinae, a substantial number of *RBP-J *mutant retinal cells survived.

### Alteration of cell types in the mature *RBP-J *deficient retina

**Figure 3:**
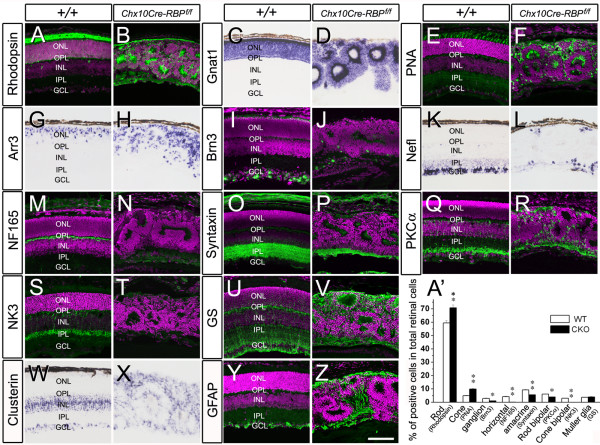
**Alteration of cell types in mature *RBP-J *deficient retinae**. Immunostaining (green) and *in situ *hybridization performed on P21 sections from *Chx10Cre-RBP*^*f*/*f *^and wild-type retinae. **(A-H) **Compared to control retinae, both rhodopsin^+ ^and *Gnat1*^+ ^rod photoreceptor cells, and PNA^+ ^and *Arr3*^+ ^cone photoreceptor cells are significantly increased in *RBP-J *deficient retinae. **(I-L) **Brn3^+ ^and *Nefl*^+ ^ganglion cells are greatly decreased in *RBP-J *deficient retinae compared to wild-type. **(M-T) **Interneurons, including NF165^+ ^horizontal cells (M, N), syntaxin^+ ^amacrine cells (O, P), PKCα^+ ^rod bipolar cells (Q, R), and NK3^+ ^cone bipolar cells (S, T) are all decreased in *RBP-J *deficient retinae, relative to wild-type controls. **(U-Z) **Numbers of glutamine synthetase (GS)^+ ^and *clusterin*^+ ^Müller glial cells in *Chx10Cre-RBP*^*f*/*f *^retinae are comparable to those in wild-type retinae, although GFAP^+ ^endfeet of Müller glial cells are increased in the deficient retina. **(A') **Comparison of percentages of Rhodopsin^+^, PNA^+^, Brn3^+^, NF165^+^, syntaxin^+^, PKCα^+^, NK3^+ ^and GS^+ ^cells in the total population of retinal cells between *Chx10Cre-RBP*^*f*/*f *^and wild-type retinae (*P < 0.05, **P < 0.01). GCL, ganglion cell layer; INL, inner nuclear layer; IPL, inner plexiform layer; ONL, outer nuclear layer; OPL, outer plexiform layer. Hoechst counterstaining is shown in magenta. Scale bar, 100 μm.

The mature neural retina is composed of seven distinct types of cells partitioned into stereotypic layers. To determine whether retinal cell types were altered in the absence of RBP-J, we performed immunolabeling and *in situ *hybridization of various retinal cell markers in the mature *RBP-J *deficient retinae. We found that rhodopsin^+ ^and *Gnat1*^+ ^rod photoreceptors(Davidson et al., 1994;Chen et al., 2005b), as well as PNA^+ ^and *Arr3*^+ ^cone photoreceptors [22] were remarkably increased in *Chx10Cre-RBP*^*f*/*f *^retinae compared to P21 controls (Figure 3A-H and 3A'). On the other hand, the number of ganglion cells was greatly reduced, as shown by Brn3 immunostaining [31] and by *Nefl in situ *hybridization (Figure 3I-L and 3A'). Similarly, immunostaining of NF165 and syntaxin [2] indicated that the horizontal and amacrine interneuron populations were also dramatically reduced (Figure 3M-P and 3A'). In addition, PKCα^+ ^rod bipolar cells were decreased in number, while NK3^+ ^cone bipolar cells [32] were nearly completely lost in *RBP-J *deficient retinae (Figure 3Q-T and 3A'). On the other hand, the number of Müller glial cells was unchanged, as indicated by the labeling of glutamine synthetase and *clusterin *(Figure 3U-X and 3A'), although GFAP immunostaining showing the endfeet of Müller glial cells [33] appeared to be increased relative to controls (Figure 3Y and 3Z). It should be noted that *Chx10Cre-RBP*^*f*/+ ^mice showed neither reduction of eye size nor changes of retinal cell types compared to wild-type controls, suggesting there is no haplo-insufficiency effect of *RBP-J *gene in mammalian retinal development. Taken together, these results show that the relative population size of photoreceptors is increased, while that of other neuronal cell types is decrease, in the case of Müller glial cells, unchanged in the mature *RBP-J *deficient retina.

### Deletion of *RBP-J *leads to overproduction of ganglion cells during early retinogenesis

**Figure 4:**
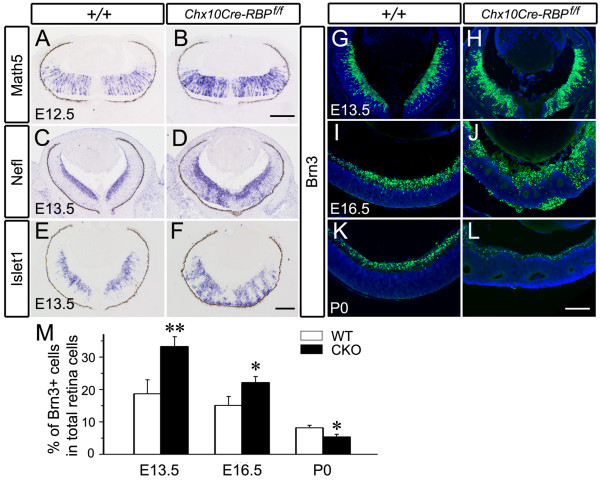
**Up-regulation of ganglion cell differentiation-related genes and increase of ganglion cells in early retinogenesis of *Chx10Cre-RBP*^*f*/*f *^mice**. **(A, B) **Expression of the proneural gene *Math5 *is increased in *RBP-J *deficient retinae compared to wild-type retinae at E12.5. **(C-F) **The expression domains of *Nefl *and *islet1*, two ganglion cell markers, are enlarged in *RBP-J *deficient retinae (D, F) compared with wild-type controls at E13.5 (C, E). **(G-L) **Immunostaining for Brn3 reveals an increase in the number of ganglion cells at E13.5 (G, H) and E16.5 (I, J), but a decrease at P0 (K, L) in *RBP-J *deficient retinae (H, J, L) relative to wild-type controls (G, I, K). Hoechst counterstaining is shown in blue. **(M) **Comparison of percentages of Brn3^+ ^cells in the total population of retinal cells between *Chx10Cre-RBP*^*f*/*f *^and wild-type retinae (*P < 0.05, **P < 0.01). Scale bars, 100 μm.

Canonical Notch signaling inhibits neurogenesis [3,34], and therefore the blockade of Notch pathway in the retina is expected to result in enhanced production of neuronal cells, especially of the first-born ganglion cells [8,35]. However, the population of ganglion cells was decreased in the mature *Chx10Cre-RBP*^*f*/*f *^retinae (Figure 3). To gain more insights into this inconsistency, we focused our examination of *RBP-J *deficient retinae at stages prior to E14.5, at which abnormal cell death was first detected (Figure 2). The differentiation of ganglion cells was accelerated in *Chx10Cre-RBP*^*f*/*f *^embryos at E12.5 and E13.5, as shown by the up-regulation of *Math5*, which promotes the ganglion cell fate [34], and two ganglion cell marker genes, *Nefl *and *Islet1 *[9] (Figure 4A-F). Furthermore, we found that the proportion of Brn3^+ ^ganglion cells in the total Hoechst-counterstained retinal cells was increased in *Chx10Cre-RBP*^*f*/*f *^retinae at E13.5 and E16.5 (Figure 4H, J and 4M), but was reduced by P0 (Figure 4L and 4M). Thus, inactivation of *RBP-J *in retina leads to overproduction of ganglion cells at embryonic stages, but does not persist postnatally. This phenotype is distinct from that of *Notch1 *deficient retinae in which ganglion cells are reduced at both embryonic and postnatal stages [9,10].

### Precocious differentiation and ultimate reduction of interneurons in *Chx10Cre-RBP*^*f*/*f *^retinae

**Figure 5:**
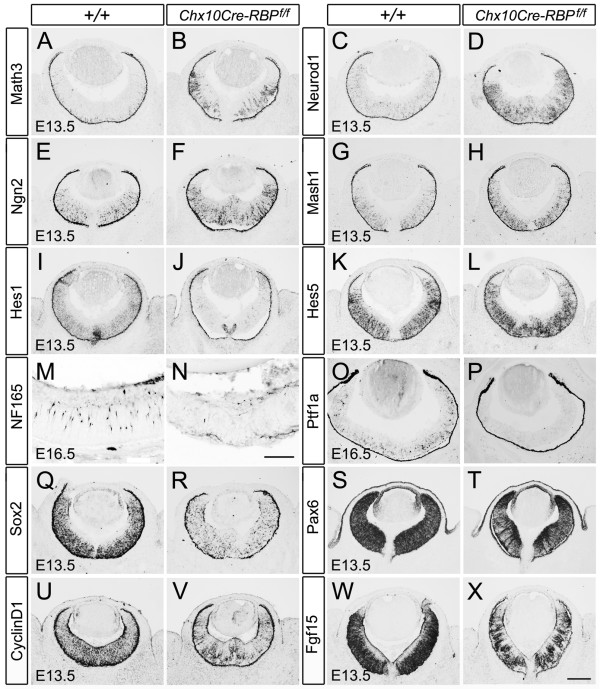
**Up-regulation of interneurons differentiation-related genes but decrease of interneurons in *Chx10Cre-RBP*^*f*/*f *^retinae at embryonic stages**. **(A-H) **Expression of *Math3 *(A, B), *Neurod1 *(C, D), *Ngn2 *(E, F) and *Mash1 *(G, H) are enhanced in *RBP-J *deficient retinae (B, D, F, H) compared to wild-type retinae (A, C, E, G) at E13.5. **(I-L) ***Hes1 *expression is reduced but *Hes5 *expression is unchanged in *RBP-J *deficient retinae (J, L) relative to wild-type controls (I, K). **(M-P) **NF165-immunoreactive cells and *Ptf1a*-expressing cells are reduced in *RBP-J *deficient retina at E16.5 (N, P) compared with wild-type controls (M, O). **(Q-X) **Expression of *Sox2 *(Q, R), *Pax6 *(S, T), *CyclinD1 *(U, V), and *Fgf15 *(W, X) are all decreased in *RBP-J *deficient retinae (R, T, V, X) compared with wide-type controls at E13.5 (Q, S, U, W). Scale bars, A-L, Q-X, 100 μm, M-P, 100 μm.

Interneurons are underrepresented in mature *Chx10Cre-RBP*^*f*/*f *^retinae (Figure 3). To determine whether there was a concomitant change in the generation of interneurons, we stained *Chx10Cre-RBP*^*f*/*f *^retinae for the bHLH proneural gene *Math3 *that promotes the differentiation of all three interneuron cell types (horizontal, amacrine and bipolar cells) [25,34]. At E13.5, the expression domain of *Math3 *was restricted to the outer part (close to the pigmented epithelium) in control retinae, whereas it was remarkably expanded into the inner part of *Chx10Cre-RBP*^*f*/*f *^retinae (Figure 5A and 5B). Furthermore, the bHLH proneural genes *Neurod1*, which promotes amacrine cell and photoreceptor fates [34,36], *Mash1*, which is involved in the differentiation of bipolar cells and photoreceptors [37], and *Ngn2 *[38] were all up-regulated in *RBP-J *deficient retinae at E13.5 (Figure 5C-H). We also examined the expression of Notch downstream *Hes *genes in *RBP-J *deficient retinae. We found that *Hes1 *expression was dramatically decreased in *Chx10Cre-RBP*^*f*/*f *^retinae at E13.5, when initial morphological change occurred (Figure 5I and 5J), while *Hes5 *and *Hesr2 *showed no obvious change compared to controls (Figure 5K, L and Additional file 1: Figure S4).

Although these proneural genes were up-regulated at E13.5, the population of retinal interneurons was reduced at later embryonic stages. We found that the number of horizontal cells, the first interneuron type generated, was greatly decreased at E16.5, as determined by NF165 immunostaining (Figure 5M and 5N). Postmitotic precursors of GABAergic interneurons [39-41] were decreased as well, as shown by *Ptf1a in situ *hybridization (Figure 5O and 5P). In addition, the mRNA levels of the HMG box transcription factor *Sox2 *and the homeodomain transcription factor *Pax6*, which are involved in the maintenance of RPCs [2], were also decreased at E13.5 (Figure 5Q-T). Furthermore, *Cyclin D1 *and *Fgf15 *were decreased as well (Figure 5U-X). Taken together, these results suggest that in the absence of RBP-J, RPCs likely differentiate prematurely into interneuron precursors, and the eventual reduction in the number of interneurons in later retinogenesis might be due to subsequent lowered cell proliferation and/or excessive cell death.

### Inactivation of RBP-J promotes photoreceptor specification

**Figure 6:**
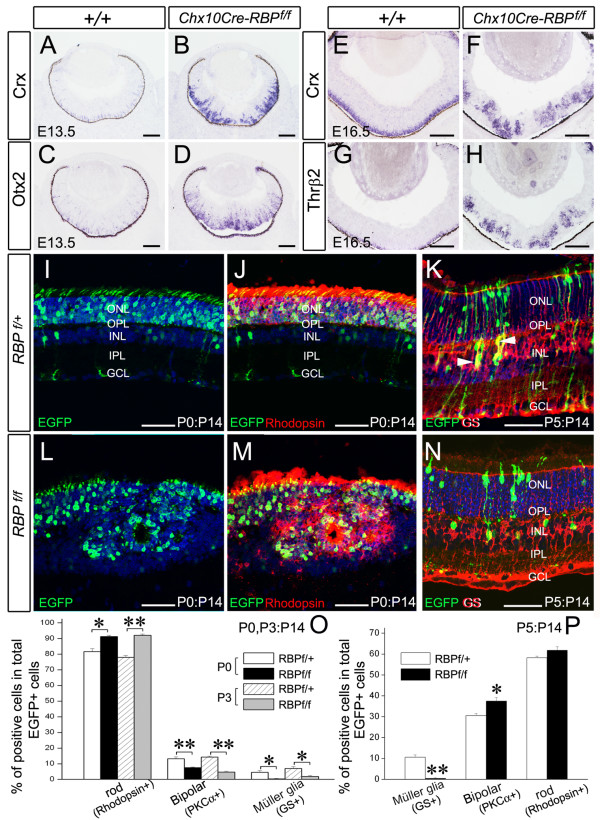
**Inactivation of *RBP-J *enhances production of photoreceptors**. **(A-D) **Expression levels of *Crx *and *Otx2*, two photoreceptor precursor markers, are significantly increased in *Chx10Cre-RBP*^*f*/*f *^retinae (B, D) compared to wild-type controls at E13.5 (A, C). **(E, F) **Enhanced *Crx *expression is also observed in *Chx10Cre-RBP*^*f*/*f *^retina (F) relative to wild-type retina at E16.5 (E). **(G, H) **Expression of *Thrβ2*, a specific marker of cone photoreceptor precursors, is also up-regulated at E16.5 in *Chx10Cre-RBP*^*f*/*f*^retina (H) relative to wild-type retina (G). **(I, J)**Expressing Cre at P0 by *in vivo *electroporation in *RBP*^*f*/+ ^retinae does not lead to apparent morphological alterations at P14. **(L, M) **Expressing Cre in *RBP*^*f*/*f *^retinae at P0 results in the appearance of rosettes, an increase in rhodopsin^+ ^photoreceptors and a decrease in bipolar and Müller glial cells at P14. **(K, N) **Inactivation of *RBP-J *by *in vivo *electroporation-induced Cre expression in *RBP*^*f*/*f *^retinae but not in in *RBP*^*f*/+ ^retinae at P5 results in a decrease of GS^+ ^Müller glial cells (arrows in K) and an increase in bipolar cells at P14. Hoechst counterstaining is shown in blue. **(O, P) **Comparison of immunoreactive cell numbers between *RBP*^*f*/+ ^and *RBP*^*f*/*f *^retinae after inactivation of *RBP-J *expression at P0 and P3 (O) and P5 (P). * *P *< 0.05, ** *P *< 0.01. Scale bars, 100 μm.

The transcription factors *Otx2 *and *Crx *are essential for photoreceptor specification and terminal differentiation, respectively [42]. We examined their expression in *RBP-J *deficient retina. In normal retinae, *Otx2 *and *Crx *were expressed only in the outer part at E13.5, but their expression domains were expanded into the inner part of *Chx10Cre-RBP*^*f*/*f *^retinae (Figure 6A-D). Furthermore, while *Crx*, *Otx2 *and the cone precursor-specific gene *Thrβ2 *were restricted to the outermost layer in control retinae at E16.5, their expression was found throughout *Chx10Cre-RBP*^*f*/*f *^retinae, and specifically enriched at the center of rosettes (Figure 6E-H, data not shown).

Unlike cone photoreceptors, no obvious increase of rod precursors were detected as measured by the expression of *NR2e3 *in *Chx10Cre-RBP*^*f*/*f *^retinae at embryonic and early postnatal stages (Additional file 1: Figure S5 and data not shown). Because rod precursors differentiate relatively late in retinogenesis, and rod photoreceptors are increased in the mature *Chx10Cre-RBP*^*f*/*f *^retinae (Figure 3), our failure to see an increase of the number of *Nr2e3*^+ ^cells might be due to possible secondary defects during retinal development. To further determine the role of *RBP-J *in rod photoreceptor specification, we performed *in vivo *electroporation of Cre in *RBP*^*f*/*f *^retinae at P0 and P3 to inactivate *RBP-J *within the peak stage of active generation of rod photoreceptors as well as bipolar cells [34]. pCAG-Cre and pCAG-EGFP plasmids were co-electroporated into the retinae of *RBP*^*f*/+^, *RBP*^*f*/*f *^and wild-type mice. EGFP^+ ^cells showed Cre immunoreactivity (see below). By P14, most electroporated RPCs in *RBP*^*f*/+ ^and wild-type retinae had differentiated into rod photoreceptors in the outer nuclear layer and, to a lesser degree, bipolar and Müller glial cells in the inner nuclear layer (Figure 6I, J and data not shown), consistent with previous data [30]. However, in *RBP*^*f*/*f *^retinae, the number of EGFP/rhodopsin-colabeled cells was obviously increased, whereas EGFP/PKC-colabeled bipolar cells and EGFP/glutamine synthetase-colabeled Müller glial cells were greatly reduced (Figure 6L, M, and data not shown). Quantitative analysis of P0- and P3-electroporation data revealed that the percentage of rhodopsin^+ ^rod photoreceptors in the total number of EGFP^+ ^cells was increased in *RBP*^*f*/*f *^retinae compared with controls (Figure 6O). Correspondingly, the percentages of PKCα^+ ^bipolar cells and glutamine synthetase^+ ^Müller glial cells in the total number of EGFP^+ ^cells were decreased (Figure 6O). These results indicate that RPCs are more biased to take on the rod photoreceptor fate in the absence of RBP-J in the retina. We also observed that the inactivation of *RBP-J *at P0 and P3 resulted in the appearance of rosettes (Figure 6L, data not shown). Thus, our data show that the deletion of *RBP-J *in RPCs during early retinogenesis results in an increase of the two types of photoreceptors, and postnatal inactivation leads to an overproduction of photoreceptors at the expense of bipolar and Müller glial cells.

### Postnatal inactivation of RBP-J at P5 also decreases Müllerglial cells

Notch signaling promotes gliogenesis [4,12]. We found that inactivating *RBP-J *at P0 and P3 impeded the generation of Müller glial cells (Figure 6), but their number was not decreased in mature *Chx10Cre-RBP*^*f*/*f *^retinae (Figure 3). To further dissect the role of RBP-J in retinal gliogenesis, we inactivated *RBP-J *by *in vivo *electroporation-induced Cre expression at P5, the stage at which Müller glial cell differentiation is mostly active [34]. In P14 *RBP*^*f*/+ ^and wild-type mice, EGFP-labeled cells were distributed in the inner nuclear layer and the photoreceptor-enriched outer nuclear layer. Furthermore, most of EGFP-labeled cells in the inner nuclear layer extended two processes to the basal and apical surfaces, the typical morphology of mature Müller glial cells (Figure 6K). In contrast, none of EGFP-labeled mutant cells in the inner nuclear layer possessed this morphological characteristic of Müller glial cells in *RBP*^*f*/*f *^retinae (Figure 6N). Glutamine synthetase immunostaining confirmed these morphological observations. Approximately 10.51 ± 1.20% of EGFP-labeled cells were glutamine synthetase^+ ^in *RBP*^*f*/+ ^retinae, compared to only 0.40 ± 0.04% in *RBP*^*f*/*f *^retinae (Figure 6K, N and 6P). The percentage of PKCα^+ ^bipolar cells in all EGFP^+ ^cells increased from 30.52 ± 1.01% in *RBP*^*f*/+ ^retina to 37.40 ± 1.74% in *RBP*^*f*/*f *^retina. In addition, the percentage of rhodopsin^+ ^rod photoreceptors changed from 58.24 ± 0.72% in *RBP*^*f*/+ ^retina to 61.74 ± 1.81% in *RBP*^*f*/*f *^retina, albeit not with statistical significance (Figure 6P). These results indicate that deleting *RBP-J *at P5 impairs the generation of Müller glial cells.

### RBP-J participates in retinal lamination through apical β-catenin expression

**Figure 7:**
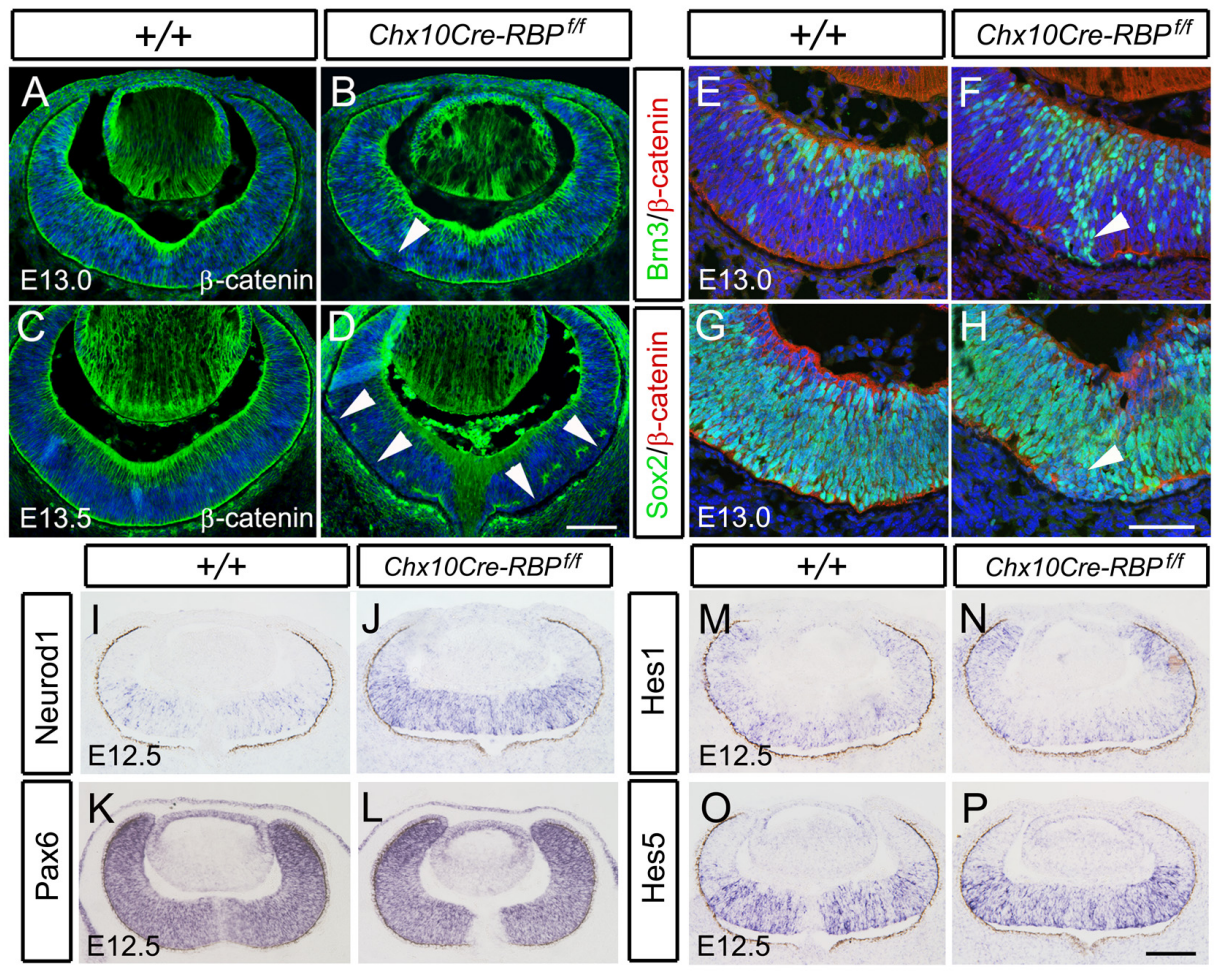
**Apical disturbance of β-catenin expression and localization is accompanied by precocious differentiation of RPCs in *Chx10Cre-RBP*^*f*/*f *^retinae**. **(A-D) **In wild-type retinae at E13.0 and E13.5, β-catenin expression is enriched along the apical surface of the retina (A, C). In the E13.0 *RBP-J *deficient retinae, however, expression is discontinuous (arrowhead in B), and the extents lacking β-catenin are enlarged at E13.5 (arrowheads in D). **(E-H) **Brn3^+ ^ganglion cells are increased (arrowhead in F), but Sox2^+ ^RPCs (arrowhead in H) are decreased in the gap region lacking β-catenin immunoreactivity in *RBP-J *deficient retinae at E13.0, as compared with wild-type controls (E, G). **(I-P) **Expression of *Neurod1 *is up-regulated (J), but that of *Pax6 *(L), *Hes1 *(N) and *Hes5 *(P) is not altered in *RBP-J *deficient retinae relative to wild-type controls at E12.5 (I, K, M, O). Scale bars, 100 μm.

In addition to abnormal development of the different retinal cell types, *Chx10Cre-RBP*^*f*/*f *^mice also exhibited retinal lamination defects. It has been shown that conditional deletion of β-catenin disrupts lamination and causes many rosette-like structures to form [17], and that Notch activity regulates the cytoplasmic level of β-catenin in vertebrates [43] and invertebrates [44,45]. We thus examined the expression of β-catenin at E13.0 and E13.5 by immunohistochemistry. In normal retinae, β-catenin was enriched at both apical and basal surfaces (Figure 7A and 7C). However, in *RBP-J *deficient retinae, β-catenin immunoreactivity was absent from the apical surface in a few regions at E13.0, and these gaps were expanded and increased in number at E13.5 (Figure 7B and 7D). Counterstaining with Hoechst at E13.5 revealed that apical extents lacking β-catenin overlayed the rosettes (Figure 7B and 7D).

**Figure 8:**
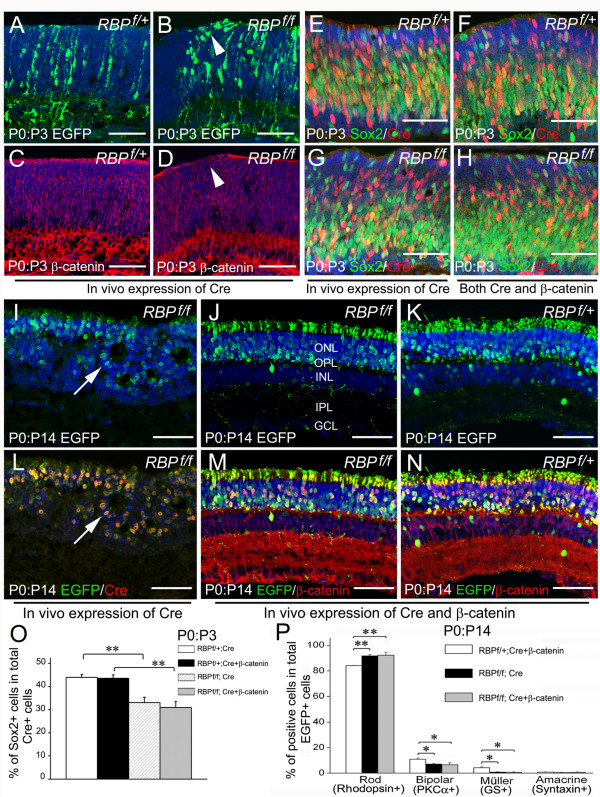
**Supplying β-catenin restores retinal laminated structures in RBP-J deficient retina**. **(A-D) ***In vivo *electroporation-induced Cre expression in *RBP*^*f*/*f*^retinae at P0 results in a loss of β-catenin at the apical surface of the retina at P3 (arrowheads in B, D), whereas this manipulation does not lead to detectable changes in β-catenin distribution in *RBP*^*f*/+ ^retinae (A, C). **(E-H, O) **Expressing Cre (G) and both of Cre and β-catenin (H) in *RBP*^*f*/*f *^retinae reduces the proportion of Sox2^+ ^cells in the total Cre^+ ^cells compared with *RBP*^*f*/+ ^retinae electroporated with Cre (E) and both of Cre and β-catenin (F). Statistical comparison of the percentages of Sox2^+ ^cells in the total Cre^+ ^cells is shown in (O). **P < 0.01. **(I, L) **Expression of Cre in *RBP*^*f*/*f *^retina at P0 results in the appearance of rosettes (arrows) at P14. **(J, M) **Overexpression of β-catenin prevents the formation of rosette-like structures in Cre-electroporated *RBP*^*f*/*f *^retinae. **(K, N) **Overexpression of β-catenin and Cre in *RBP*^*f*/+ ^retinae. Note that there are few EGFP^+ ^cells in the inner nuclear layer (INL) and the vast majority of EGFP^+ ^cells are located in the outer nuclear layer (ONL) in Cre and β-catenin expressing *RBP*^*f*/*f *^retinae (J), as compared with Cre and β-catenin misexpressing *RBP*^*f*/+ ^retinae (K). **(P) **Comparison of rod photoreceptors, bipolar cells, amacrine cells and Müller glia cells at P14 between *RBP*^*f*/+ ^and *RBP*^*f*/*f *^retinae after *in vivo *misexpression of Cre and β-catenin at P0 (* *P *< 0.05, ** *P *< 0.01). Hoechst counterstaining (blue) is shown to reveal the retinal layers. Scale bars, 100 μm.

The precocious differentiation of RPCs already appeared at E13.5 (Figure 4 and 6), coexisted with the disturbance of apical β-catenin expression. To gain more insights into their relationship, we examined the expression of cell markers in *RBP-J *deficient retinae at E13.0, the earliest stage when β-catenin gap was observed. Double-immunostaining showed that Brn3^+ ^ganglion cells were increased locally in the retinal regions lacking β-catenin. Accordingly, Sox2^+ ^RPCs were initially decreased within these gaps, as compared with those in normal β-catenin expressing regions (Figure 7E-H). At E12.5, prior to the β-catenin expression defects, *Math5 *and *Neurod1 *were up-regulated (Figure 4A and 4B; Figure 7I and 7J), while the level of *Math3*, *Mash1*, *Pax6*, *Hes1 *and *Hes5 *showed no differences compared with controls (Figure 8K-P, data not shown).

We further inactivated *RBP-J *at P0 by electroporating Cre into *RBP*^*f*/*f *^retinae. We observed a similar phenotype: β-catenin expression at the apical surface was lost by P3 in areas proximal to clusters of EGFP^+ ^cells (Figure 8A-D). Expression of Cre in *RBP*^*f*/+ ^or wild-type retinae, in contrast, did not affect β-catenin expression (Figure 8A and 8C, data not shown). Although the proportion of rhodopsin^+ ^cells in the total population of EGFP^+ ^cells was not altered (Additional file 1: Figure S6) at P3, Sox2^+ ^RPCs in the total population of Cre^+ ^cells was decreased in the electroporated regions of *RBP*^*f*/*f *^retinae, as compared with Cre-electroporated *RBP*^*f*/+ ^or wild-type retinae (Figure 8E, G and 8O, data not shown).

To further clarify that the loss of apical β-catenin expression contributes to defective lamination in *RBP-J *deficient retina, we co-transfected P0 *RBP*^*f*/+ ^and *RBP*^*f*/*f *^retinae with pCAG-Cre, pCAG-β-catenin and pCAG-EGFP. EGFP-expressing cells showed enhanced accumulation of cytoplasmic β-catenin at P14 (Figure 8M and 8N). Interestingly, the expression of β-catenin in *RBP-J *deficient retinae prevented the formation of rosettes, restoring lamination to normality in transfected areas of *RBP*^*f*/*f *^retinae (Figure 8J and 8M). However, expression of β-catenin did not rescue the retinal cell specification defects, as Sox2^+ ^RPCs were decreased at P3, rod photoreceptors were present in excess at P14, and the number of bipolar cells was still reduced at the same stage (Figure 8H, O and 8P). Co-transfection of β-catenin and Cre at P0 did not cause any detectable morphological changes or cell type change in *RBP*^*f*/+ ^or wild-type retinae at P3 (Figure 8E, F and 8O) and P14 (Figure 8K, N and 8P, data not shown). These results indicate that *RBP-J*-modulated β-catenin expression in the apical retina is necessary for the formation of retinal laminar structures.

## Discussion

In the present study, the transcription factor RBP-J, which integrates signals from the four mammalian Notch receptors, was specifically inactivated in mouse RPCs, thereby circumventing potential functional redundancy among Notch receptors during retinal development. We found that disruption of RBP-J in early retinogenesis resulted in overproduction of ganglion cells and photoreceptors at the expense of retinal interneurons, while postnatal deletion of *RBP-J *at P0/P3 promoted the generation of rod photoreceptors at the expense of bipolar and Müller glial cells, and deletion at P5 enhances the production of bipolar cells at the expanse of Müller glial cells. These results suggest that RBP-J-mediated signaling controls cell specification and differentiation in retinogenesis in a stepwise manner. Furthermore, the formation of rosette-like structures in *RBP-J *deficient retina is likely a consequence of disturbed apical β-catenin expression, because restoring β-catenin expression rescued the lamination defects. In summary, our results show that RBP-J signaling plays multiple roles during retinal cell specification, differentiation and lamination.

### Roles of RBP-J in retinal neurogenesis

Among the six neuronal cell types, only the population of photoreceptors was found to be increased in both embryonic and mature *Chx10Cre-RBP*^*f*/*f *^retinae (Figure 3 and 6). The result also get support from the observation that postnatal inactivation of RBP-J in the retina at P0 and P3 also leads to an overproduction of photoreceptors and this occurs at the expense of bipolar and Müller glial cells (Figure 6). Therefore, it is likely that in the absence of RBP-J, RPCs are biased to take on photoreceptor fate in the retinogenesis. This is consistent with the recent finding that the suppression of Notch signaling promotes ES cell-derived RPCs to differentiate into *Crx*^+ ^photoreceptor precursors [46].

In *Notch1 *deficient retina, only cone photoreceptors are increased [9,10], whereas both two types of photoreceptors as well as ganglion cells are overproduced in *RBP-J *deficient retinae (Figure 3 and 4). These differences might underscore compensatory influences of other Notch receptors expressed in *Notch1 *deficient retinae. For instance, *Notch3*, which is also expressed in the neural retina (Additional file 1: Figure S1), could inhibit the expression of specification- and/or differentiation-related genes and thereby prevent the superfluous generation of ganglion cells and rod photoreceptors(Brown et al., 2001; Wang et al., 2001). The differences between the *Notch1 *and *RBP-J *deficient retinae also suggest that different Notch receptors may regulate the generation of distinct types of retinal cells.

During early stages of retinogenesis, the number of ganglion cells was increased in *Chx10Cre-RBP*^*f*/*f *^retinae, but ultimately reduced by P0 (Figure 3 and 4). This may be explained by an increase in cell death at later stages of retinogenesis in the absence of RBP-J. On the other hand, the expression of several bHLH proneuronal genes that are known to promote retinal interneuron differentiation is up-regulated at E13.5, but the number of interneurons is decreased at embryonic and postnatal stages (Figure 3 and 5). This suggests that the rate at which RPCs differentiate into retinal interneurons is accelerated in the early retinogenesis, and this precocious differentiation, which in turn leads to the depletion of their precursor pool, together with elevated levels of apoptosis and the preferential generation of photoreceptors, would lead to a net decrease of these neurons in *RBP-J *deficient retinae. This could happen in the Notch dependent or independent manner. A recent study has shown that RBP-J forms a complex with the transcription factor Ptf1a in spinal cord to promote the differentiation of GABAergic interneurons, independent of the canonical Notch pathway [47]. Interestingly, *Ptf1a*, which is also expressed in horizontal and amacrine cell precursors and is involved in the differentiation of the two types of interneuron [39-41], is down-regulated in *RBP-J *deficient retinae. Further experiments are needed to determine if RBP-J and Ptf1a interact in retinal precursors and if the RBP-J-Ptf1a complexes regulate horizontal and amacrine cell differentiation.

### Gliogenesis in *RBP-J *deficient retinae

Notch signaling promotes glial cell differentiation. A recent study has shown that knocking out *RBP-J *in the dorsal root ganglia results in the loss of glia cells [48], but we did not observe obvious change of Müller glial population size in the mature *Chx10Cre-RBP*^*f*/*f *^retinae (Figure 3). However, postnatal inactivation of *RBP-J *at P0, P3 and P5 led to a decrease in the number of glia cells in the retina (Figure 6), suggesting that RBP-J acts as a positive regulator of retinal gliogenesis. No obvious change of Müller glia cells in the mature *RBP-J *deficient retinae is likely to be a consequence of neuronal loss and/or global distorted lamination, which may lead to reactive gliogenesis. On the other hand, *Hes5 *and *Hesr2*, two Notch-RBP-J effectors that promote retinal gliogenesis [12,28], remained unchanged in the *RBP-J *deficient retina (Figure 5 and Additional file 1: Figure S4). It is therefore possible that the sustained expression of *Hes5 *and *Hesr2 *contributes to normal generation of Müller glial cells in *RBP-J *deficient retina. This finding also suggests the existence of a RBP-J independent pathway that activates or maintains *Hes5 *and *Hesr2 *expression in the *RBP-J *deficient retina.

### RBP-J regulates retinal lamination via maintaining apical β-catenin expression

Lamination defects always appear in mouse retinae when Notch signaling is impaired. For example, *Notch1 *deficient and *Hes1 *mutant retinae both display similarly distorted morphologies, including the appearance of numerous rosette-like structures [9,11,49]. Three main factors are thought to contribute to retinal lamination: the retinal pigmented epithelium, Müller glial cells, and cell adhesion among RPCs [50]. Lamination defects in *Chx10Cre-RBP*^*f*/*f *^retinae are most likely due to defective cell adhesion among RPCs, because *Chx10Cre *is not expressed in the pigmented epithelium, and furthermore, the defects precede Müller glial cell differentiation. RPC adherens junctions include the homophilic adhesion molecule N-cadherin and the intracellular binding partner β-catenin, which links the cytoplasmic domain of N-cadherin to the actin cytoskeleton. Eliminating N-cadherin expression in *Zebrafish *disrupts retinal lamination[51], while conditional inactivation of β-catenin in the mouse retina also results in severe retinal lamination defect[17].

Adherens junctions form at both the apical and basal surfaces of the developing retina, where the endfeet of RPCs are anchored, with the apical junctions are especially important for retinal lamination [17,50]. We found that the morphological changes in *RBP-J *deficient retinae were correlated with the disturbance of apical expression of β-catenin, and that overexpression of β-catenin could rescue the lamination phenotype (Figure 7 and 8). Thus, normal expression of β-catenin in the retina requires RBP-J, while defective β-catenin expression leads to the formation of rosette-like structure in *RBP-J *deficient retina. Notch activity has been shown to regulate the cytoplasmic level of β-catenin [43-45], and RBP-J has been found to bind to β-catenin in mouse neural precursor cells [52]. Thus it could be interesting in future studies to explore if such interactions also exist in RPCs and if loss of apical β-catenin is caused directly by deficiency of RBP-J. On the other hand, since the up-regulation of proneural genes *Math5 *and *Neurod1 *precedes the disturbance of apical expression of β-catenin, and an increase of Brn3^+ ^ganglion cells and a decrease of Sox2^+ ^RPCs are present in the retinal region with defective apical expression of β-catenin (Figure 7), it is possible that this disturbance of β-catenin expression is due to the change of bHLH expression profiles in the absence of RBP-J. Further studies are needed to elucidate whether the up-regulation of *Math5 *and *Neurod1 *leads to defective expression of β-catenin in RBP-J deficient retina.

Supplying β-catenin by *in vivo *electroporation could rescue the lamination defects but not precocious differentiation or cell type mispecification in *RBP-J *deficient retina, while overexpression of β-catenin in wild-type and *RBP*^*f*/+ ^retinae did not affect the differentiation of RPCs (Figure 8). These results suggest that the restoration of lamination defect by β-catenin is not achieved by inhibition of precocious differentiation of RPCs in *RBP-J *deficient retina. In addition, conditional deletion of β-catenin in the retina leads to abnormal lamination, without affecting either cell specification or differentiation [17]. Therefore, it seems most likely that the role of β-catenin in retinal lamination is independent of retinal cell differentiation.

## Conclusions

In our present study, we conditionally inactivated transcription factor RBP-J which integrates all Notch receptor signals in mouse retina, and found that RBP-J mediated Notch signaling inhibits ganglion cell and photoreceptor differentiation, and promotes Müller glial cell differentiation during retinogenesis. In addition, RBP-J regulates retinal lamination via maintaining apical β-catenin expression. Altogether, our results indicate that RBP-J mediated Notch signaling not only governs cell-type differentiation, but also participates in cellular organization during retinal development, which ensure the exquisite process of retinogenesis.

## Competing interests

The authors declare that they have no competing interests.

## Authors' contributions

The authors have made the following declarations about their contributions: Conceived and designed the experiments: HH, YQD. Performed the experiments: MHZ, ZP. Analyzed the data: MHZ, MS, FG. Wrote the manuscript: YQD, HH, MHZ. All authors read and approved the final manuscript.

## Supplementary Material

Additional file 1 **Zheng et al Supplementary materials**. The file contains Figure S1-S6 and their figure legends. Click here for file

## References

[B1] Chow RL Lang RA Early eye development in vertebrates Ann Rev Cell Dev Biol 2001 17 255 296 10.1146/annurev.cellbio.17.1.255 11687490

[B2] Marquardt T Gruss P Generating neuronal diversity in the retina: one for nearly all Trends Neurosci 2002 25 32 38 10.1016/S0166-2236(00)02028-2 11801336

[B3] Louvi A Artavanis-Tsakonas S Notch signalling in vertebrate neural development Nat Rev Neurosci 2006 7 93 102 10.1038/nrn1847 16429119

[B4] Furukawa T Mukherjee S Bao ZZ Morrow EM Cepko CL Rax, Hes1, and notch1 promote the formation of Muller glia by postnatal retinal progenitor cells Neuron 2000 26 383 394 10.1016/S0896-6273(00)81171-X 10839357

[B5] Han H Tanigaki K Yamamoto N Kuroda K Yoshimoto M Nakahata T Ikuta K Honjo T Inducible gene knockout of transcription factor recombination signal binding protein-J reveals its essential role in T versus B lineage decision Int Immunol 2002 14 637 645 10.1093/intimm/dxf030 12039915

[B6] Bray SJ Notch signalling: a simple pathway becomes complex Nat Rev Mol 2006 7 678 689 10.1038/nrm2009 16921404

[B7] Dorsky RI Rapaport DH Harris WA Xotch inhibits cell differentiation in the xenopus retina Neuron 1995 14 487 496 10.1016/0896-6273(95)90305-4 7695895

[B8] Austin CP Feldman DE Ida JA Cepko CL Vertebrate retinal ganglion cells are selected from competent progenitors by the action of Notch Development 1995 121 3637 3650 8582277 10.1242/dev.121.11.3637

[B9] Jadhav AP Mason HA Cepko CL Notch1 inhibits photoreceptor production in the developing mammalian retina Development 2006 133 913 923 10.1242/dev.02245 16452096

[B10] Yaron O Farhy C Marquardt T Applebury M Shery-Padan R Notch1 functions to suppress cone-photoreceptor fate specification in the developing mouse retina Development 2006 133 1367 1378 10.1242/dev.02311 16510501

[B11] Takatsuka K Hatakeyama J Bessho Y Kageyama R Roles of the bHLH gene Hes1 in retinal morphogenesis Brain Res 2004 1004 148 155 10.1016/j.brainres.2004.01.045 15033430

[B12] Hojo M Ohtsuka T Hashimoto N Gradwohl G Guillemot F Kageyama R Glial cell fate specification modulated by the bHLH gene Hes5 in mouse retina Development 2000 127 2515 2522 10821751 10.1242/dev.127.12.2515

[B13] Hinds JW Hinds PL Early ganglion cell differentiation in the mouse retina: an electron microscopic analysis utilizing serial sections Dev Biol 1974 37 381 416 10.1016/0012-1606(74)90156-0 4826283

[B14] Hinds JW Hinds PL Differentiation of photoreceptors and horizontal cells in the embryonic mouse retina: an electron microscopic, serial section analysis J Comp Neurol 1979 187 495 511 10.1002/cne.901870303 489789

[B15] Neumann CJ Pattern formation in the zebrafish retina Semin Cell Dev Biol 2001 12 485 490 10.1006/scdb.2001.0272 11735384

[B16] Poggi L Zolessi FR Harris WA Time-lapse analysis of retinal differentiation Curr Opin Cell Biol 2005 17 676 681 10.1016/j.ceb.2005.09.004 16226448

[B17] Fu X Sun H Klein WH Mu X beta-catenin is essential for lamination but not neurogenesis in mouse retinal development Dev Biol 2006 299 424 437 10.1016/j.ydbio.2006.08.015 16959241 PMC3385515

[B18] Rowan S Cepko CL Genetic analysis of the homeodomain transcription factor Chx10 in the retina using a novel multifunctional BAC transgenic mouse reporter Dev Biol 2004 271 388 402 10.1016/j.ydbio.2004.03.039 15223342

[B19] Dai JX Han HL Tian M Cap J Xiu BJ Song MM Huang Y Xu XL Ding YQ Xu L Enhanced contextual fear memory in central serotonin-deficient mice Proc Natl Acad Sci USA 2008 105 11981 11986 10.1073/pnas.0801329105 18695238 PMC2575315

[B20] Soriano P Generalized lacZ expression with the ROSA26 Cre reporter strain Nat Genet 1999 21 70 71 10.1038/5007 9916792

[B21] Guo C Qiu HY Huang Y Chen H Yang RQ Chen SD Johson RL Chen ZF Ding YQ Lmx1b is essential for Fgf8 and Wnt1 expression in the isthmic organizer during tectum and cerebellum development in mice Development 2007 134 317 325 10.1242/dev.02745 17166916

[B22] Chen J Rattner A Nathans J The rod photoreceptor-specific nuclear receptor Nr2e3 represses transcription of multiple cone-specific genes J Neurosci 2005 25 118 129 10.1523/JNEUROSCI.3571-04.2005 15634773 PMC6725199

[B23] Blackshaw S Harpavat S Trimarchi J Cai L Huang H Kuo WP Weber G Lee K Fraioli RE Cho SH Yung R Asch E Ohno-Machado L Wong WH Cepko CL Genomic analysis of mouse retinal development PLoS Biol 2004 2 E247 10.1371/journal.pbio.0020247 15226823 PMC439783

[B24] Le TT Wroblewski E Patel S Riesenberg AN Brown NL Math5 is required for both early retinal neuron differentiation and cell cycle progression Dev Biol 2006 295 764 778 10.1016/j.ydbio.2006.03.055 16690048

[B25] Inoue T Hojo M Bessho Y Tano Y Lee JE Kageyama R Math3 and NeuroD regulate amacrine cell fate specification in the retina Development 2002 129 831 842 11861467 10.1242/dev.129.4.831

[B26] Baek JH Hatakeyama J Sakamoto S Ohtsuka T Kageyama R Persistent and high levels of Hes1 expression regulate boundary formation in the developing central nervous system Development 2006 133 2467 2476 10.1242/dev.02403 16728479

[B27] Taranova OV Magness ST Fagan BM Wu Y Surzenko N Hutton SR Pevny LH SOX2 is a dose-dependent regulator of retinal neural progenitor competence Genes Dev 2006 20 1187 1202 10.1101/gad.1407906 16651659 PMC1472477

[B28] Satow T Bae SK Inoue T Inoue C Miyoshi G Tomita K Bessho Y Hashimoto N Kageyama R The basic Helix-Loop-Helix gene hesr2 promotes gliogenesis in mouse retina J Neurosci 2001 21 1265 1273 11160397 10.1523/JNEUROSCI.21-04-01265.2001 PMC6762251

[B29] Peng YR He S Marie H Zeng SY Ma J Tan ZJ Lee SY Malenka RC Yu X Coordinated changes in dendritic arborization and synaptic strength during neural circuit development Neuron 2009 15 71 84 10.1016/j.neuron.2008.11.015 PMC2713111 19146814

[B30] Matsuda T Cepko CL Inaugural Article: Electroporation and RNA interference in the rodent retina in vivo and in vitro Proc Natl Acad Sci USA 2004 101 16 22 10.1073/pnas.2235688100 14603031 PMC314130

[B31] Liu W Khare SL Liang X Peters MA Liu X Cepko CL Xiang M All Brn3 genes can promote retinal ganglion cell differentiation in the chick Development 2000 127 3237 3247 10887080 10.1242/dev.127.15.3237

[B32] Haverkamp S Ghosh KK Hirano AA Wässle H Immunocytochemical description of five bipolar cell types of the mouse retina J Comp Neurol 2003 455 463 476 10.1002/cne.10491 12508320 PMC2834891

[B33] Chen H Weber AJ Expression of glial fibrillary acidic protein and glutamine synthetase by Muller cells after optic nerve damage and intravitreal application of brain-derived neurotrophic factor Glia 2002 38 115 125 10.1002/glia.10061 11948805

[B34] Hatakeyama J Kageyama R Retinal cell fate determination and bHLH factors Semin Cell Dev Biol 2004 15 83 89 10.1016/j.semcdb.2003.09.005 15036211

[B35] Nelson BR Gumuscu B Hartman BH Reh TA Notch activity is downregulated just prior to retinal ganglion cell differentiation Dev Neurosci 2006 28 128 141 10.1159/000090759 16508310

[B36] Morrow EM Furukawa T Lee JE Cepko CL NeuroD regulates multiple functions in the developing neural retina in rodent Development 1999 126 23 36 9834183 10.1242/dev.126.1.23

[B37] Tomita K Moriyoshi K Nakanishi S Guillemot F Kageyama R Mammalian achaete-scute and atonal homologs regulate neuronal versus glial fate determination in the central nervous system EMBO J 2000 19 5460 5472 10.1093/emboj/19.20.5460 11032813 PMC314003

[B38] Ma W Wang SZ The final fates of neurogenin2-expressing cells include all major neuron types in the mouse retina Mol Cell Neurosci 2006 31 463 469 10.1016/j.mcn.2005.10.018 16364654 PMC1876733

[B39] Dullin JP Locker M Robach M Henningfeld KA Parain K Afelik S Pieler T Perron M Ptf1a triggers GABAergic neuronal cell fates in the retina BMC Dev Biol 2007 7 110 119 10.1186/1471-213X-7-110 17910758 PMC2212653

[B40] Nakhai H Sel S Favor J Mendoza-Torres L Paulsen F Duncker GI Schmid RM Ptf1a is essential for the differentiation of GABAergic and glycinergic amacrine cells and horizontal cells in the mouse retina Development 2007 134 1151 1160 10.1242/dev.02781 17301087

[B41] Fujitani Y Fujitani S Luo H Qiu F Burlison J Long Q Kawaguchi Y Edlund H MacDonald RJ Furukawa T Fujikado T Magnuson MA Xiang M Wright CV Ptf1a determines horizontal and amacrine cell fates during mouse retinal development Development 2006 133 4439 4450 10.1242/dev.02598 17075007

[B42] Nishida A Furukawa A Koike C Tano Y Aizawa S Matsuo I Furukawa T Otx2 homeobox gene controls retinal photoreceptor cell fate and pineal gland development Nat Neurosci 2003 6 1255 1263 10.1038/nn1155 14625556

[B43] Nicolas M Wolfer A Raj K Kummer JA Mill P van Noort M Hui CC Clevers H Dotto GP Radtke F Notch1 functions as a tumor suppressor in mouse skin Nat Genet 2003 33 416 421 10.1038/ng1099 12590261

[B44] Hayward P Kalmar T Arias AM Wnt/Notch signalling and information processing during development Development 2008 135 411 424 10.1242/dev.000505 18192283

[B45] Hayward P Brennan K Sanders P Balayo T DasGupta R Perrimon N Martinez Arias A Notch modulates Wnt signalling by associating with Armadillo/β-catenin and regulating its transcriptional activity Development 2005 132 1819 1830 10.1242/dev.01724 15772135 PMC2500123

[B46] Osakada F Ikeda H Mandai M Wataya T Watanabe K Yoshimura N Akaike A Sasai Y Takahashi M Toward the generation of rod and cone photoreceptors from mouse, monkey and human embryonic stem cells Nat Biotech 2008 26 215 224 10.1038/nbt1384 18246062

[B47] Hori K Cholewa-Waclaw J Nakada Y Glasgow SM Masui T Henke RM Wildner H Martarelli B Beres TM Epstein JA Magnuson MA Macdonald RJ Birchmeier C Johnson JE A nonclassical bHLH Rbpj transcription factor complex is required for specification of GABAergic neurons independent of Notch signaling Genes Dev 2008 22 166 178 10.1101/gad.1628008 18198335 PMC2192752

[B48] Taylor MK Yeager K Morrison SJ Physiological Notch signaling promotes gliogenesis in the developing peripheral and central nervous systems Development 2007 134 2435 2447 10.1242/dev.005520 17537790 PMC2653864

[B49] Tomita K Ishibashi M Nakahara K Ang SL Nakanishi S Guillemot F Kageyama R Mammalian hairy and enhancer of split homolog 1 regulates differentiation of retinal neurons and is essential for eye morphogenesis Neuron 1996 16 723 734 10.1016/S0896-6273(00)80093-8 8607991

[B50] Koike C Nishida A Akimoto K Nakaya MA Noda T Ohno S Furukawa T Function of atypical protein kinase C in differentiating photoreceptors is required for proper lamination of mouse retina J Neurosci 2005 25 10290 10298 10.1523/JNEUROSCI.3657-05.2005 16267237 PMC6725782

[B51] Erdmann B Kirsch FP Rathjen FG Moré MI N-cadherin is essential for retinal lamination in the zebrafish Dev Dyn 2003 226 570 577 10.1002/dvdy.10266 12619142

[B52] Shimizu T Kagawa T Inoue T Nonaka A Takada S Aburatani H Taga T Stablilized beta-catenin functions through ECF/LEF proteins and the Notch/RBPJ complex to promote proliferation and suppress differentiation of neural precursor cell Mol Cell Biol 2008 28 7427 7441 10.1128/MCB.01962-07 18852283 PMC2593432

