# Supplementary material for: The transcription factor RBP-J is essential for retinal cell differentiation and lamination
Source: Mol Brain. 2009 Dec 18;2:38. doi: 10.1186/1756-6606-2-38 (PMC2804697; doi:10.1186/1756-6606-2-38)
Supplement: Additional file 1 — Zheng et al Supplementary materials. The file contains Figure S1-S6 and their figure legends. [file 1756-6606-2-38-S1.PDF]

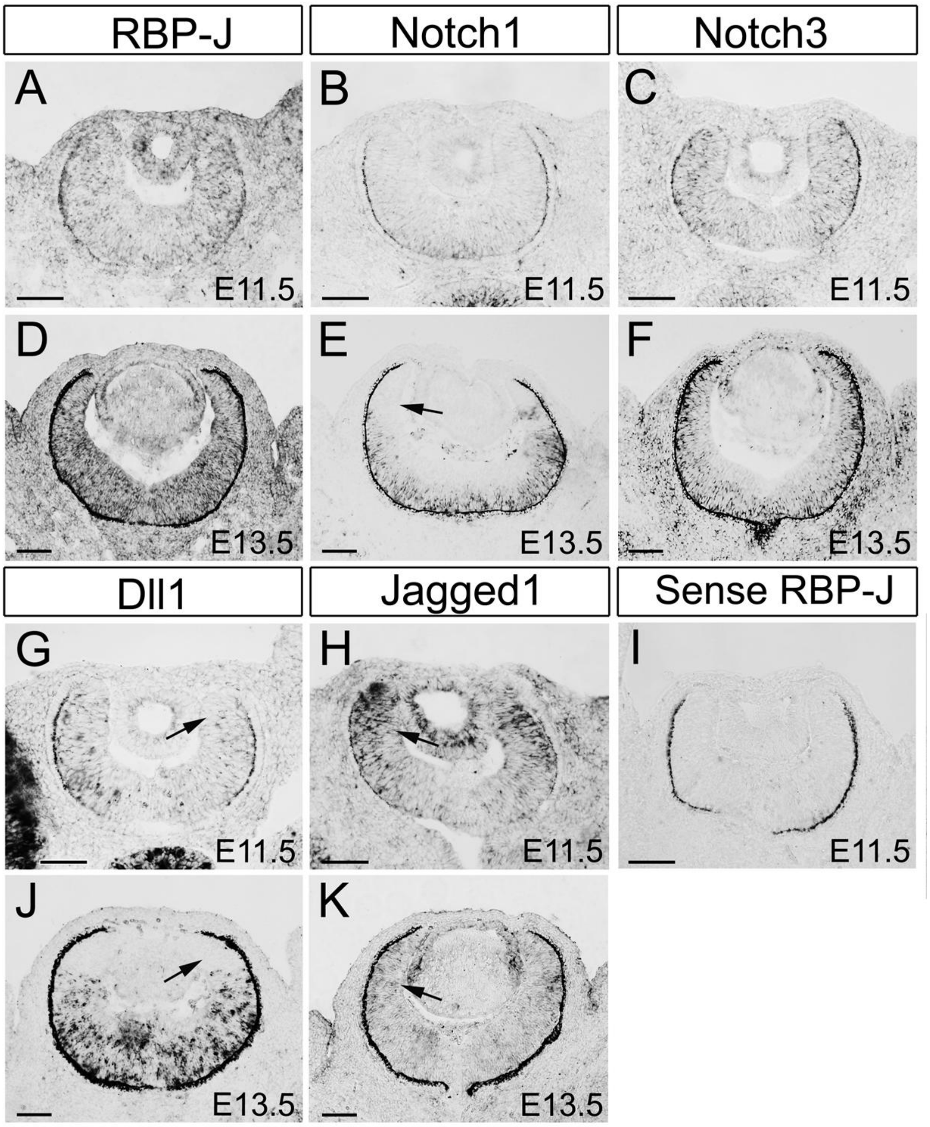

Figure S1. Expression of *RBP-J* and Notch pathway-related genes in the retina. **(A,D)** *RBP-J* mRNA in the retina at E11.5 (A) and E13.5 (D). **(B-F)** *Notch1* was expressed in the central part of the retina (B, E), while the *Notch3* expression domain covered the entire retina (C, F) at E11.5 (B, C) and E13.5 (E, F). **(G-K)** *Dll1* and *Jagged1* showed somewhat complementary patterns of expression, with *Dll1* mostly in the central areas of the retina (G, J) and *Jagged1* enriched in the periphery (H, K) at E11.5 (G, H) and E13.5 (J, K). **(I)** Sense *RBP-J* probe detects no *in situ* signal. Arrowheads (E, G, H, J, K) point to peripheral region of the retina. Scale bars, 100

μm.

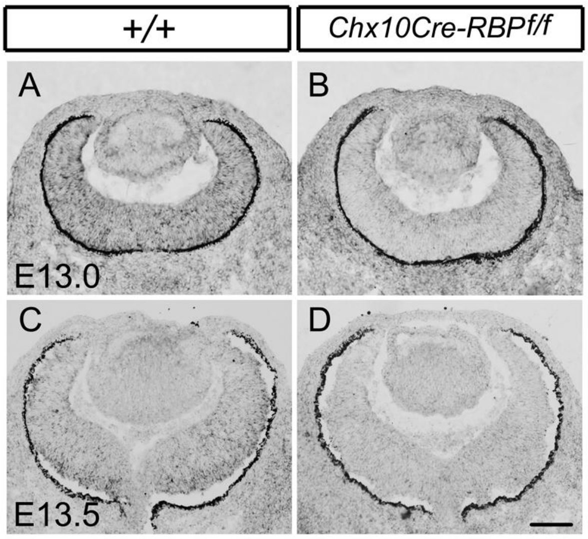

Figure S2. Effective inactivation of *RBP-J* in the retinae of *Chx10Cre-RBP*$^{f/f}$ mice. (A-D) *In situ* hybridization shows that *RBP-J* expression is reduced at E13.0, and almost completely lost by E13.5. Scale bar, 100 μm.

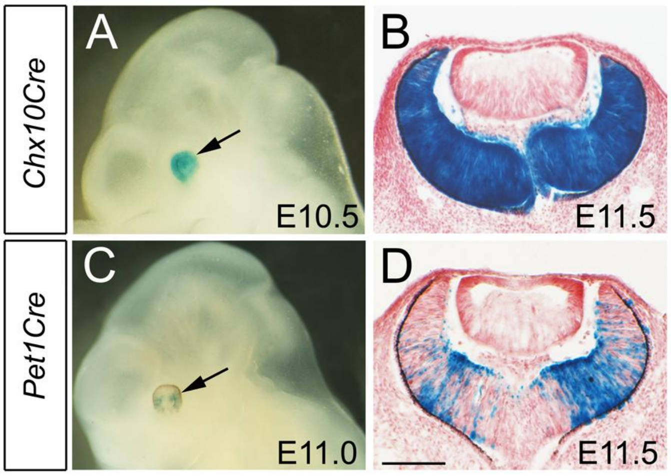

Figure S3. Comparision of Cre activity in the retina between *Chx10Cre* and *Pet1Cre* transgenic mice. (A-D) Cre activity initiates in the retina at approximately E10.5 in *Chx10Cre* mice (arrow in A), and at E11.0 in *Pet1Cre* mice (arrow in C), as shown by X-gal staining. Note that at E11.5, robust X-gal staining is observed throughout the retina of *Chx10Cre* mice (B), whereas X-gal staining in retinae of

E11.5 *Pet1Cre* mice is neither as strong, nor as uniform (D). Scale bar, 100 μm.

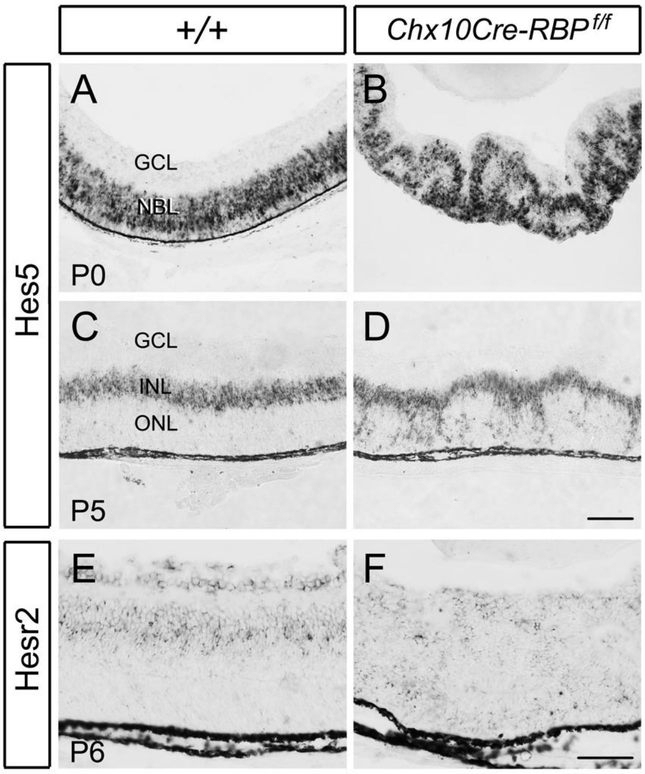

Figure S4. Expression of *Hes5* and *Hesr2* is maintained in *Chx10Cre-RBP*$^{f/f}$ retinae. (A-D) The mRNA level of *Hes5* is comparable in *Chx10Cre-RBP*$^{f/f}$ retinae to wild-type controls at P0 and P5. **(E,F)** Expression level of *Hesr2* remains similar in *RBP-J* deficient retinae at P6 relative to age-matched wild-type controls. GCL, ganglion cell layer; NBL, neuroblastic layer; INL, inner nuclear layer: ONL, outer nuclear layer. Scale bars, 100 μm.

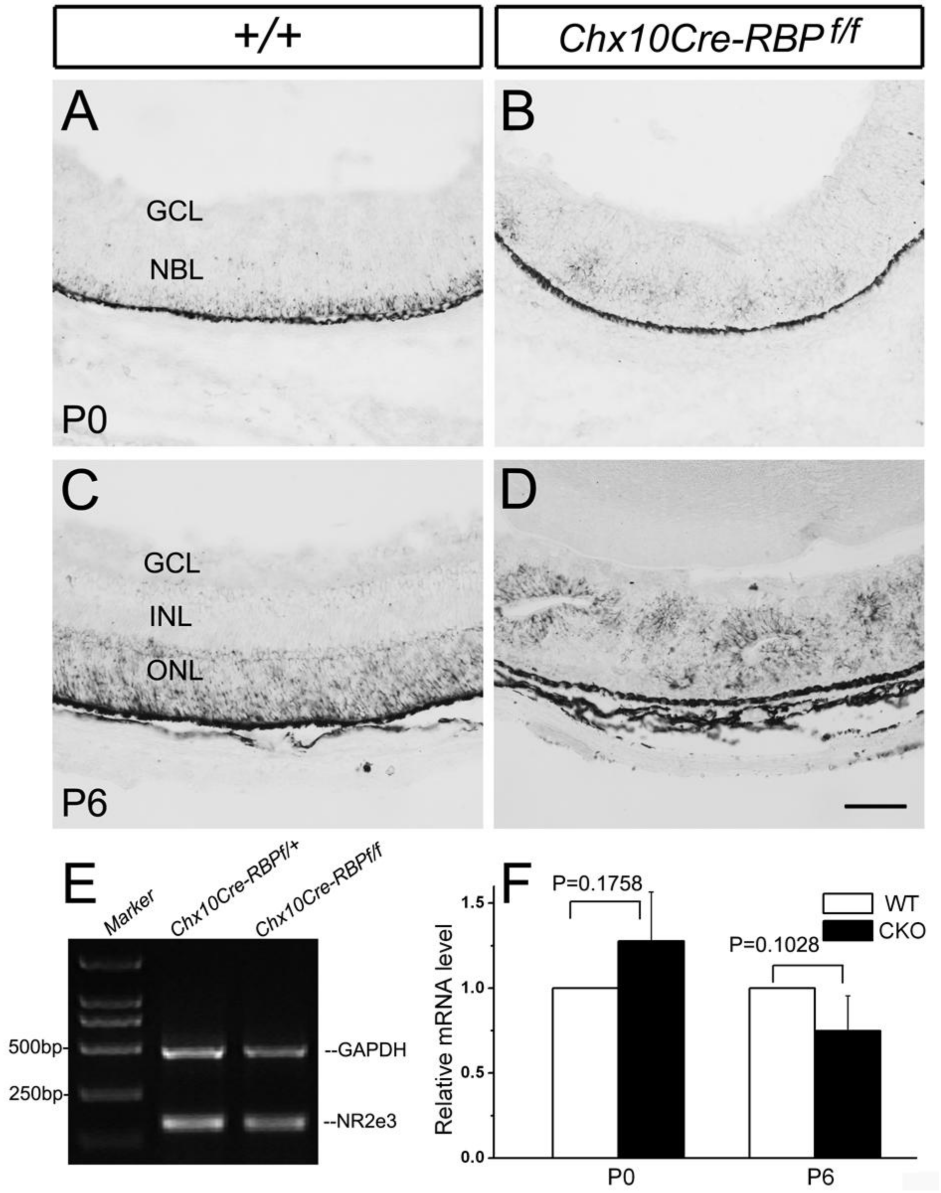

Figure S5. Expression of *NR2e3* appears to be unchanged in *Chx10Cre-RBP* $^{f/f}$ retinae. (A-D) Expression level of *NR2e3* remains similar in *RBP-J* deficient retinae at P0 (B) and P6 (D) compared to age-matched wild-type controls (A, C). **(E,F)** RT-PCR results show the specifically amplified *NR2e3* DNA fragment at 150 bp, and this pair of primer was used in real-time PCR. qPCR results indicate similar expression levels of NR2e3 in wild-type and *RBP-J* deficient retinae. GCL, ganglion cell layer; NBL, neuroblastic layer; INL, inner nuclear layer: ONL, outer nuclear layer. Scale bar, 100 μm.

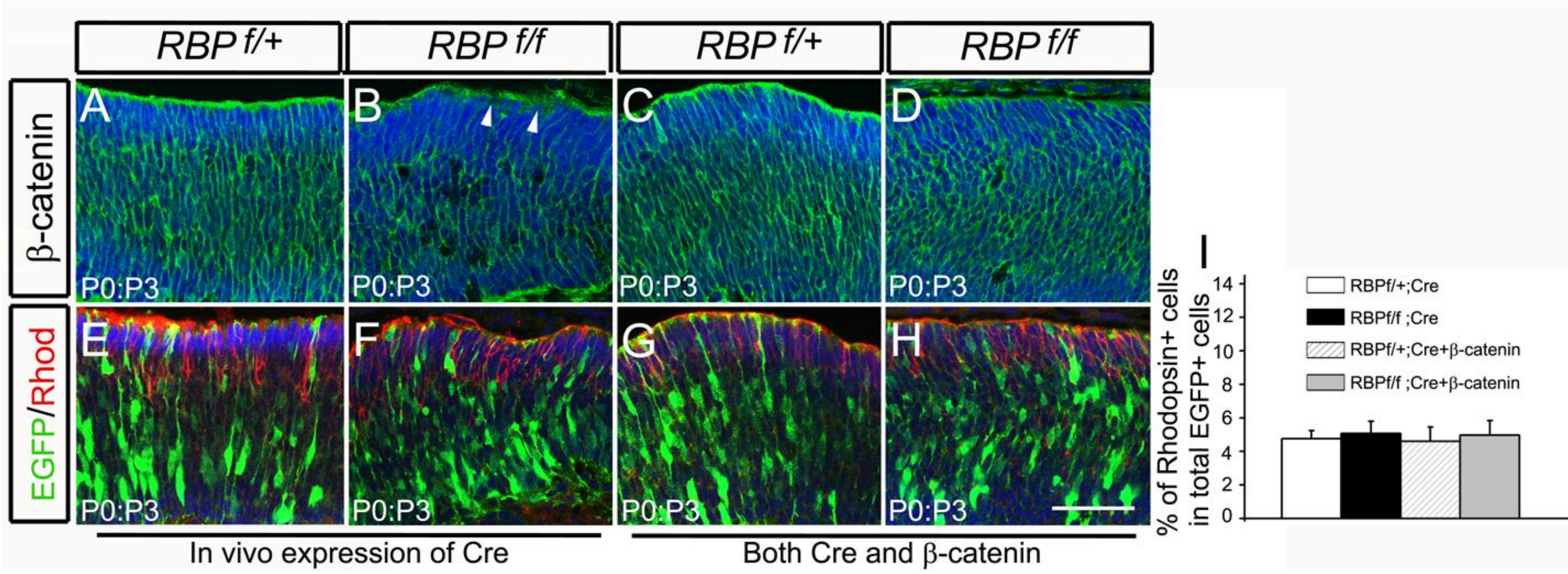

Figure S6. Cell type analysis of P0 electroporated retina at P3. (A-D) Apical β-catenin expression is disturbed in Cre-electroporated $RBP^{f/f}$ retinae (arrowhead in B) at P3 compared with that in $RBP^{f/+}$ retinae (A). Co-electroporation of β-catenin with Cre in $RBP^{f/f}$ retinae rescues the apical disturbance although the accumulation of β-catenin protein is still weak at this stage (D). **(E-H)** Double labeling on adjacent sections shows that a few EGFP+ cells differentiate into rhodopsin^{+} photoreceptors in both electroporated $RBP^{f/+}$ and $RBP^{f/f}$ retinae at P3. Hoechst counterstaining is shown in blue. **(I)** Statistic result shows no significant difference between the percentages of rhodopsin^{+} photoreceptors in the total of EGFP^{+} cells in electroporated $RBP^{f/f}$ and $RBP^{f/+}$retinae. Scale bar, 100 μm.
